# Socioeconomic status and other factors associated with HIV status among OVC in Democratic Republic of Congo (DRC)

**DOI:** 10.3389/fpubh.2022.912787

**Published:** 2022-10-03

**Authors:** Gulzar H. Shah, Gina D. Etheredge, Lievain Maluantesa, Kristie C. Waterfield, Osaremhen Ikhile, Elodie Engetele, Astrid Mulenga, Alice Tabala, Bernard Bossiky

**Affiliations:** ^1^Jiann-Ping Hsu College of Public Health, Georgia Southern University, Statesboro, GA, United States; ^2^FHI, Washington DC, CA, United States; ^3^FHI 360, Kinshasa, Congo; ^4^ICAP, Columbia University, New York, NY, United States; ^5^National Multisectoral HIV/AIDS program (PNMLS), HIV Program, Presidency of DRC, Kinshasa, Congo

**Keywords:** HIV, DRC, orphans, vulnerable children, socioeconomic status

## Abstract

**Background:**

Orphans and vulnerable children (OVC) are a high-risk group for HIV infection, particularly in Sub-Saharan Africa.

**Purpose:**

This study aims to portray the socioeconomic profile of OVC and examine the association of household and parent/guardian characteristics with the HIV status of OVC.

**Methods:**

For this quantitative retrospective study, we obtained data from ICAP/DRC for a total of 1,624 OVC from households enrolled for social, financial, and clinical services between January 2017 and April 2020 in two provinces of the Democratic Republic of Congo, Haut-Katanga and Kinshasa. We computed descriptive statistics for OVC and their parents' or guardians' characteristics. We used the chi-square test to determine bivariate associations of the predictor variables with the dichotomous dependent variable, HIV positivity status. To analyze the association between these independent variables and the dichotomous dependent variable HIV status after controlling for other covariates, we performed firth's logistic regression.

**Results:**

Of the OVC included in this study, 18% were orphans, and 10.9% were HIV+. The chi-square analysis showed that among parents/guardians that were HIV+, a significantly lower proportion of OVC (11.7%) were HIV+ rather than HIV- (26.3%). In contrast, for parents/guardians with HIV- status, 9.0% of OVC were HIV-negative, and 11.7% of OVC were OVC+. The firth's logistic regression also showed the adjusted odds of HIV+ status were significantly lower for OVC with parents/guardians having HIV+ status themselves (AOR, 0.335; 95% CI, 0.171–0.656) compared with HIV-negative parents/guardians. The adjusted odds of HIV+ status were significantly lower for OVC with a monthly household income of < $30 (AOR, 0.421; 95% CI, 0.202–0.877) compared with OVC with a monthly household income > $30.

**Conclusions:**

Our results suggest that, with the exception of a few household and parent/guardian characteristics, the risk of HIV+ status is prevalent across all groups of OVC within this study, which is consistent with the existing body of evidence showing that OVC are in general vulnerable to HIV infection. With a notable proportion of children who are single or double orphans in DRC, HIV+ OVC constitute a high-risk group that merits customized HIV services. The findings of this study provide data-driven scientific evidence to guide such customization of HIV services.

## Introduction

Human immunodeficiency virus (HIV) infection control programs need scientific evidence on HIV risk factors in vulnerable populations such as orphans and vulnerable children (OVC) to eliminate new HIV infections and to customize HIV prevention, treatment, and management services (1). Public health and non-profit organizations continue to set and pursue aggressive targets for the elimination of HIV/AIDS; therefore, understanding the profile of OVC is critical, given that they have a significantly higher risk of HIV infection than other children (2). The premature deaths of people living with HIV (PLHIV), many of whom are parents, directly exacerbate the vulnerability of their children (3, 4). In 2019, there were approximately 405,000 PLHIV in the Democratic Republic of the Congo (DRC). Of those, approximately 51,000 were OVC (5). In the DRC, orphans are defined as children (under 18 years of age) that have lost either one or both parents to war and disease (6, 7). Vulnerable children are people under 15 years of age who are prone to abuse, violence, and economic hardship because of their circumstances of birth or immediate environment. They are often deprived of basic needs, care, and protection and are disadvantaged compared to their peers (8–10).

The HIV epidemic continues to be a global health problem, specifically in resource-limited countries in Sub-Saharan Africa, such as the DRC. This remains particularly true for pediatric HIV patients, some of whom are OVC; over 90 percent of all pediatric HIV patients reside in Sub-Saharan Africa (11). PEPFAR/DRC has been continually intensifying its efforts in epidemic control and strengthening services to support the National AIDS Control Program (PNLS), which moves them closer to the UNAIDS' 95-95-95 targets in the provinces of Haut-Katanga and Lualaba (5). Among their highest priorities are their efforts to support OVCs and their families with a comprehensive set of services, including health, economic, and educational services, especially concerning strengthening the care continuum (5). There is strong evidence that such customized interventions specifically designed for high-risk subgroups produce positive outcomes and are cost-effective (12).

Vulnerability and other factors such as orphan status, isolation and stress, social stigma, and hardship are detrimental to OVC's physical and emotional health (2) and increase the risk of HIV infection and treatment failure (13). The level of social support patients greatly influences adult and adolescent health outcomes from the “network of family, friends, neighbors, and community members that is available in times of need to give psychological, physical, and financial help.” Among the OVC in resource-limited countries, the amount of social support is either very limited or non-existent (11, 14, 15). The burden of HIV infection encompasses medical, social, and economic issues. It thus presents a very complex range of psychosocial challenges for not only the patients but also for their caregivers. These challenges become even more complex and cumbersome when the patients are pediatric patients (14). Recent studies have found that pediatric patients with low socioeconomic status and thus reduced access to transportation, education, and economic opportunities are less likely to adhere to antiretroviral therapy (ART) (15).

Parents and caregivers significantly impact children's and youths' treatment adherence (16). In their 2017 study, Nichols, Steinmetz, and Paintsil found that parents or caregivers would not disclose to the child their HIV status if it were positive, fearing the negative psychological impact children may experience after knowing their status (11). Since 2017, health personnel in the DRC have had guidelines for managing HIV disclosure to children with the consent and cooperation of the parents/guardians (17). Unfortunately, non-disclosure leads to children's inability to understand the reasons for taking medication or the risks of certain behaviors, which may result in non-adherence to treatment and preventative care (11, 18). Additionally, households with parents or caregivers that are also HIV+ experience an additional negative socioeconomic impact. Some of the children in these households, especially OVCs, are forced to abandon their education and work very early to meet their basic needs (15). Furthermore, OVCs that are taken care of by a sick parent or caregiver are more likely to engage in unsafe or risky behaviors (19).

The DRC's HIV+ OVC face the same challenges as those in other resource-limited Sub-Saharan countries. Currently, over 700,000 children in the DRC have been left orphaned due to HIV (20). With the majority of the population living in extreme poverty and the slowed economic growth of recent years (21), the DRC has an increased need to understand how household socioeconomic status and parent/guardian characteristics impact the health status (HIV status) of OVC. Evidence-based decision-making in HIV interventions is especially critical for OVC. Research studies on OVC's HIV status elsewhere show that the socioeconomic status of households and caregivers' demographic and clinical characteristics are among significant risk factors, but such studies in the DRC are scant (2, 22–24). To contribute to such scientific evidence, we have two goals in the current research: (a) to portray the profile of OVC in two provinces of DRC and (b) to explore risk factors for HIV+ status in OVC. These risk factors include characteristics of OVC and socioeconomic characteristics of the households and parents/guardians.

## Methods

### Data

Based on secondary data, this quantitative retrospective study analyzed de-identified individual-level programmatic records obtained from ICAP/DRC for 1,624 OVC enrolled between January 2017 and April 2020 in two provinces of the Democratic Republic of Congo, Haut-Katanga (*n* = 175), and Kinshasa (*n* = 1,449). This study focused on OVC as the non-OVC data were unavailable in this database. Per PEPFAR definitions, the age range for OVC is from 0 to 17 years. The source of the OVC dataset was household records, created from several different household files linked through household identification numbers. The data for these files came from household members' screening and assessment for eligibility and enrollment in various social, financial, and clinical services and from the monitoring of each household beneficiary's services. Household eligibility was assessed based on the presence of one or more of the following: (a) HIV+ children/adolescents newly started on ART, (b) HIV+ children or adolescents suspected of treatment failure, (c) children exposed to HIV who were <2 years of age, (d) children or adolescents living with an HIV+ adult at an advanced stage of the disease, and (e) high-risk teenage girls. High-risk teenage girls have an older sex partner, multiple sex partners, and/or adolescent sex workers. This high-risk group also included sexually exploited or abused adolescent girls. This dataset included both male and female OVC enrolled in the system, regardless of HIV status. HIV+ children who have already been on ART and had no treatment failure were not included in this database per the HIV program decision.

### Measures

#### Dependent variable—HIV status of OVC

The dependent variable OVC HIV status had two attributes, HIV-negative (coded as 0) and HIV+ (coded as 1). The proportion of OVC for whom the HIV status was not declared was relatively small in the final dataset (i.e., 31 or 1.9%), so they were excluded from the multivariable analyses. To ascertain the extent to which this exclusion may have caused any bias, we used the chi-square to see if those with HIV status declared vs. undeclared differed by OVC characteristics. There were small though statistically non-significant differences by age and sex; the differences were statistically significant by urban health zones and formal schooling.

#### Independent variables

Three sets of independent variables were included in the analysis. The first set consisted of child characteristics. The OVC *age at the time of enrollment* was computed by calculating the difference between the date of birth and the date of enrollment and subsequently grouped into four categories: <5 years, 5 to <10 years, 10 to < 15 years, and 15 years or older. *A child's sex* was a dichotomous variable with attributes of both males and females. Whether the *child is in school* and the *child's handicap status* were both dichotomous variables with yes and no categories, as noted in the casework database.

The second set of independent variables comprised guardian/parent and household characteristics. The *main source of drinking water* originally had three categories – (a) private hand pumps, (b) public taps, drilling, rainwater, well/protected source, and catchment scheme, and (c) river, stream, lake, pond, well, or unprotected spring. This variable was coded as dichotomy by combining (a) and (b) above. *Toilet type* was a dichotomous variable with “no toilet/latrine” and “toilet with or without flush” as two attributes. The household having a monthly income of < $30 was recorded as yes or no. Both variables, *sex of the parent/guardian* and *parent's/guardian's HIV status*, were dichotomous with the attributes of male or female and HIV-negative or HIV+, respectively. The variable a*ge of the parent/guardian* was coded as <40 years of age, 40–49 years of age, 50 years or older, and not reported. The third set of characteristics consisted of facility characteristics, one of which was included in the analysis, the *rurality or urbanicity of the health zone*, coded as “rural or semi-rural” and “urban”.

### Analytical methods

We computed descriptive statistics such as frequencies and percentages after stratification by OVC orphan status to describe the characteristics of OVC and their parents or guardians. We examined the bivariate associations between the categorical independent variables and the dichotomous dependent variable OVC HIV status using the chi-square test and a *p*-value of 0.05 or lower to determine statistical significance. To examine the association between these independent variables and the dichotomous dependent variable HIV status after controlling for other covariates, we performed firth's logistic (FL) regression to compute three separate FL models, first for the OVC characteristics as covariates, the second with parent/guardian or household socioeconomic characteristics, and the third one for the facility characteristics. Firth's logistic regression was deemed appropriate to reduce bias in the estimation of maximum likelihood (ML) coefficients resulting from small samples or extremely disproportionate binary outcomes (e.g., the model overfitting) resulting from extremely disproportionate attribute distribution of the outcome variable—HIV status (HIV+, 163 OVC vs. 1,338 HIV-) (25). Puhr et al. (2017) describe how firth's penalization in logistic regression reduces bias in the estimation of rare events (25). We performed all the analyses for this study using Stata 15 (StataCorp, LLC, College Station, TX) (26). Georgia Southern University's Institutional Review Board (IRB) approved the study under project protocol number H l9260.

## Results

Orphans constituted 18% of the OVC included in this study (Table 1). One-in-10 (10.9%) were HIV+. The mean age at enrollment was 9.3 years. Twenty-two percent of OVC were <5 years old, 29.7% were ages 5 to <10 years, 34.5% were aged 10 to < 15 years, and the remaining 13.7% were 15 years or older. Over half, or 51.6%, were females, and 2.8% of all OVCs had some disability or handicap. Only 32.5% of the OVC were in school, 56.7% were not, and the current enrollment status for the remaining 10.8% was unknown.

**Table 1:** Descriptive statistics for OVC, stratified by orphan status, Jan. 2017 to Apr. 2020.

**Child, parent/guardian, and facility characteristics**	**Child is an orphan**						**All OVC**	
	**Yes**		**No**		**Undeclared**		
	**No**.	**%**	**No**.	**%**	**No**.	**%**	**No**.	**%**
**HIV results**								
HIV-	200	67.6%	708	84.6%	430	87.6%	1338	82.4%
HIV+	69	23.3%	59	7.0%	35	7.1%	163	10.0%
Not Reported	27	9.1%	70	8.4%	26	5.3%	123	7.6%
**Age of child**								
< 5 year of age	65	22.0%	208	24.9%	86	17.5%	359	22.1%
5–9.9 years of age	93	31.4%	248	29.6%	142	28.9%	483	29.7%
10–14 years of age	88	29.7%	275	32.9%	197	40.1%	560	34.5%
15 years or older	50	16.9%	106	12.7%	66	13.4%	222	13.7%
**Child's sex**								
Female	150	50.8%	428	51.2%	258	52.7%	836	51.5%
Male	145	49.2%	408	48.8%	232	47.3%	785	48.3%
**Child in school**								
No	165	55.7%	521	62.2%	235	47.9%	921	56.7%
Yes	95	32.1%	248	29.6%	185	37.7%	528	32.5%
Not reported	36	12.2%	68	8.1%	71	14.5%	175	10.8%
**Child disabled**								
No	224	75.7%	563	67.3%	231	47.0%	1018	62.7%
Yes	11	3.7%	26	3.1%	9	1.8%	46	2.8%
Not reported	61	20.6%	248	29.6%	251	51.1%	560	34.5%
**Main source of drinking water**							
Other source	27	11.5%	77	13.1%	32	13.3%	136	8.4%
Public taps, drilling, rainwater, well/protected source, catchment scheme	208	88.5%	512	86.9%	208	86.7%	928	57.1%
**Toilet type**								
No toilet/latrine	10	11.5%	33	13.1%	7	13.3%	50	3.1%
Toilet with flush, or latrine without flush/pit toilet	225	88.5%	556	86.9%	233	86.7%	1014	62.4%
**Household income** ** < $30/month**						
No	12	4.1%	32	3.8%	8	1.6%	52	3.2%
Yes	223	75.3%	557	66.5%	232	47.3%	1012	62.3%
Not Reported	61	20.6%	248	29.6%	251	51.1%	560	34.5%
**Sex of parent/guardian**								
Female	178	60.1%	348	41.6%	168	34.2%	694	42.7%
Male	42	14.2%	223	26.6%	64	13.0%	329	20.3%
Not reported	76	25.7%	266	31.8%	259	52.7%	601	37.0%
**HIV status of parent/guardian**							
HIV-	21	7.1%	76	9.1%	47	9.6%	144	8.9%
HIV+	73	24.7%	254	30.3%	75	15.3%	402	24.8%
Not reported	202	68.2%	506	60.5%	369	75.2%	1078	66.4%
**Child in school**								
No	5	1.7%	26	3.1%	3	0.6%	34	56.7%
Yes	31	10.5%	63	7.5%	27	5.5%	121	32.5%
Not reported	260	87.8%	748	89.4%	461	93.9%	1469	10.8%
**Urban health zone vs rural health zone**						
Rural or Semi-rural	43	14.5%	115	13.7%	42	8.6%	200	12.3%
Urban	253	85.5%	722	86.3%	449	91.4%	1424	87.7%
**All OVC**	296	18.2	837	51.5	491	30.2	1624	100.0%

HIV-, HIV-Negative; HIV+, HIV-Positive; OVC, orphans and vulnerablechildren.

Most OVC (87.2%) had access to water sources comprising public taps, drilling, rainwater, wells, or other protected sources, whereas the remaining 12.8% had another source of water such as a river, stream, lake, pond, well, or unprotected spring. The majority of OVC had a toilet with or latrine without a flush/pit toilet (62.4%), whereas 3.1% had no toilet in their homes. The majority (62.3%) of OVC households had a monthly income of <$30, whereas 3.2% had a monthly income of $30 or more; for the remaining 34.5%, the household income was unknown. Forty-three percent of the parents/guardians were female, 20.3% were male, and 37.0 had no documented sex.

One-in-four parents or current guardians were HIV+, a small proportion (i.e., 8.9%) were HIV-, and for the remaining 66.4%, their HIV status was not reported. A majority (87.7%) lived in an urban health zone, whereas the remaining 12.3% were in rural or semi-rural health zones. The mean age of the parents or guardians was 44.6 years. The age distribution of the parents or guardians showed that 20.8% were <40 years of age, 21.4% were 40–49 years of age, 20.4% were 50 years old or older, and the remaining 37.4% did not have their age documented. The stratification of these characteristics is shown in Table 1.

Bivariate associations tested with chi-square statistics, depicted in Table 2, show that the risk of HIV was uniform across OVC regardless of child characteristics, with one exception. Whether the child was in school was unknown for a much larger proportion of HIV+ children than HIV-negative, 30.7% vs. 9.1 (*P* < 0.001). There was no significant statistical difference in HIV status among OVC regardless of household socioeconomic characteristics such as type of toilet, monthly household income below $30, or the source of drinking water. The risk of HIV was no different across OVC by different parent/guardian characteristics such as age or sex. However, significant variation (*P* < 0.001) in OVC's HIV status was observed in parents/guardians' own HIV status. Among parents/guardians that were HIV+, a significantly lower proportion of OVC (11.7%) were HIV+ rather than HIV-negative (26.3%). In contrast, for parents/guardians with HIV- status, 11.7% of OVC were HIV+, and 9.0% of OVC were HIV-. For parents with unknown (i.e., unreported) HIV status, 64.7% of OVC were HIV-, and 76.7% of OVC were OVC+.

**Table 2:** Bivariate Analysis of HIV Status in OVC (Chi-Square), Jan. 2017 to Apr. 2020.

**Child, parent/guardian and facility characteristics**	**HIV Status**				P*
	**HIV-**		**HIV+**		
	**No**.	**%**	**No**.	**%**	
**Age of Child**					0.801
< 5 year of age	294	22.0%	38	23.3%	
5–9.9 years of age (in text we have 5–9)	397	29.7%	52	31.9%	
10–14 years of age	462	34.5%	50	30.7%	
15 years or older	185	13.8%	23	14.1%	
**Child sex**					0.367
Female	698	52.2%	79	48.5%	
Male	639	47.8%	84	51.5%	
**Child in School**					**< 0.001**
No	752	56.2%	75	46.0%	
Yes	464	34.7%	38	23.3%	
Not reported	122	9.1%	50	30.7%	
**Child had disability**					0.748
No	846	63.2%	108	66.3%	
Yes	37	2.8%	4	2.5%	
Not reported	455	34.0%	51	31.3%	
**Main source of drinking water**					0.240
Another source	104	11.8%	9	8.0%	
Public taps, drilling, rainwater, well/protected source, catchment scheme	779	88.2%	103	92.0%	
**Toilet type**					0.287
No toilet/latrine	36	4.1%	7	6.3%	
Toilet with or latrine without flush/pit toilet	847	95.9%	105	93.8%	
**HH monthly income** ** < $30**					0.061
$30 or more	37	2.8%	10	6.1%	
Less than $30	846	63.2%	102	62.6%	
Not reported	455	34.0%	51	31.3%	
**Sex of parent/guardian**					0.799
Female	582	43.5%	75	46.0%	
Male	272	20.3%	33	20.2%	
Not reported	484	36.2%	55	33.7%	
**Parent/Guardian HIV status**					**< 0.001**
HIV-	120	9.0%	19	11.7%	
HIV+	352	26.3%	19	11.7%	
Not reported	866	64.7%	125	76.7%	
**Parent/guardian age**					0.567
< 40 years of age	283	21.2%	42	25.8%	
40-49 years of age	299	22.3%	32	19.6%	
50 years or older	269	20.1%	31	19.0%	
Not reported	487	36.4%	58	35.6%	
**Urban vs. rural**					**0.035**
Rural or Semi-rural	140	10.5%	26	16.0%	
Urban	1198	89.5%	137	84.0%	

^*^ P indicates level of significance based on Chi-Squarestatistics.

HH, Household; HIV-, HIV-Negative; HIV+,HIV-Positive.

For further assessment of the associations based on chi-square tests, the results of firth's logistic regression are presented in Table 3. After controlling for children's characteristics such as age at the time of enrollment, sex, and handicap status, the odds of HIV+ status were significantly higher for OVC with unknown school enrollment status (adjusted odds ratio (AOR), 4.140; 95% CI, 2.745–6.244) compared with OVC not in school. The odds of HIV status were not significantly different for OVC in different sex or age categories; these characteristics are considered important in HIV patients.

**Table 3:** Firth's Logistic regression of HIV Status in OVC, Jan. 2017 to Apr. 2020.

**Characteristics**	**AOR**	**P**	**95% CI**	
			**Lower**	**Upper**
**Child characteristics**				
**Age-group (vs**. **>5 years)**				
5 to < 10 years	1.087	0.723	0.683	1.729
10 to < 15 years of age	0.963	0.874	0.604	1.533
15 years or older	1.164	0.595	0.663	2.044
**Child 's Sex (vs. Male)**				
Female	1.172	0.348	0.841	1.632
**Child in School (vs. No)**				
Yes	0.822	0.356	0.542	1.245
Not reported	4.140	**< 0.001**	2.745	6.244
**Child with disability or handicap (vs. No)**				
Yes	1.065	0.903	0.384	2.953
Not reported	1.036	0.848	0.720	1.490
**Household and parent/guardian characteristics**
**Main source of drinking water (vs. unprotected water)**				
Public taps, drilling, rainwater, and other protected sources	1.688	0.146	0.833	3.420
**Toilet type (vs. no toilet/latrine)**				
Toilet with or latrine without flush/pit toilet	0.553	0.166	0.240	1.277
**HH Monthly Income less than $30 (vs. $30 or higher)**				
Less than $30	0.421	**0.021**	0.202	0.877
**Sex of parent or guardian (vs. Female)**				
Male	0.964	0.879	0.609	1.528
Not reported	0.647	0.453	0.207	2.015
**Parent/Guardian HIV status (vs. HIV-)**				
HIV+	0.335	**0.001**	0.171	0.656
Not reported	1.169	0.581	0.670	2.041
**Parent/guardian age (vs**. ** < 40 years)**				
40–49 years of age	0.728	0.207	0.445	1.191
50 years or older	0.745	0.261	0.447	1.243
Not reported	1.307	0.578	0.508	3.359
**Facility characteristics**
**Urbanicity status (vs Rural or Semi-Rural)**				
Urban	0.608	**0.031**	0.387	0.954

This table presents results from three separate Firth's Logistic Regression Models.

CI, confidence interval; HH, Household; HIV-, HIV-Negative; HIV+,HIV-Positive.

The odds of HIV+ status were significantly lower for OVC with a monthly household income of < $30 (AOR, 0.421; 95% CI, 0.202–0.877) compared with OVC with a monthly household income of > $30, after controlling for other households and parent/guardian characteristics. The adjusted odds of HIV+ status were also significantly lower for OVC with HIV+ parents/guardians (AOR, 0.335; 95% CI, 0.171–0.656) compared with HIV-negative parents/guardians. The odds of HIV+ status were significantly lower for OVC in urban areas (AOR 0.608; 95% CI, 0.387–0.954) compared to OVC in rural and semi-rural areas.

## Discussion

HIV/AIDS prevention, care, and treatment programs rely heavily on empirical evidence to improve the quality and effectiveness of their services. To contribute to such critically needed research evidence for HIV/AIDS programs in the DRC, we, in the current study, focused on analyzing individual-level programmatic data about OVC enrolled in an HIV/AIDS program based on various criteria. The data were from clinics supported by the Centers for Disease Control and Prevention (CDC) with President's Emergency Plan for AIDS Relief (PEPFAR) funding in Haut-Katanga and Kinshasa provinces. Although the national strategic plan for the response to AIDS has always taken OVC into account and underlined its importance in several strategic areas, this study sheds light on OVC's HIV status and associated factors, providing critical practice-relevant evidence.

Our study results show that orphans constituted 18% of the OVC included in this study, of which 10.9% were HIV+. Having an orphan or a child who is not the biological child of the household head in one's home is quite prevalent in the DRC. A national survey from 2013–2014 showed that in the general population, 25% of households had children under 18 years of age who lived without their parents, and 12% of the households had single orphans (one parent deceased), whereas 2.4% of the households had double orphans (both parents deceased) (27).

These OVC are truly vulnerable as they are in poor socioeconomic conditions, with 1,012 of the 1,064 or 95% living with a household income of one dollar a day or less. This is extreme poverty relative to the international poverty line set at $1.90 daily (28). These OVC were also deprived of education because despite only 22% of these children being under the age of 5; a much larger proportion (57%) were not in school. In contrast, 78% of school-age children in the DRC are enrolled in primary school (29) and 46.17% in secondary school (30). This is consistent with a recent study in Zambia that discovered an association between higher psychosocial issues within OVC populations and the limited capacities for care due to poor socioeconomic conditions and lower academic achievements (31).

It is unclear why being in school was higher for OVC HIV+ (30.7%) vs. OVC HIV-negative (9.1%). This uncertainty about school enrollment may have been because 80% of the OVC in the database were referrals by healthcare workers because they lived in a household with one or more HIV+ adults; therefore, when someone other than the parent/guardian is reporting on the child's behalf, they may not know their school enrollment status. Household vulnerability and school dropouts would contribute to low school enrollment. These analyses revealed no variation in the proportion of HIV+ OVC by other characteristics such as age and sex, indicating that OVC of all ages and both sexes needed equal attention. Our findings of the higher proportion of HIV-negative OVC in the parent/guardian group that were themselves HIV+ are noteworthy, and several explanations can be proposed as follows: (1) HIV+ parents/guardians may have had better knowledge and skills to prevent their children or OVC from HIV transmission; (2) When extended families need to place an orphan or vulnerable child with someone other than their parents, the extended family may choose an HIV-negative parent/guardian who likely has some combination of more resources, better health, and less vulnerability vs. an HIV+ person; (3) HIV+ parents, once they know their status, may decide to not have more children to protect their children from infection and stigmatization (32, 33); and (4) DRC's successful PMTCT program, protecting HIV+ women from giving birth to an HIV infected baby likely plays a key role. These results are consistent with the findings of a recent study conducted in Tanzania and South Sudan regarding the impact of the OVC's caregivers and access to clinical care and support services for HIV+ OVC (34–36). These explanations must be substantiated with additional research, perhaps requiring qualitative or mixed methods. Our findings suggest the lower odds of HIV+ status among OVC in households <$30 may be attributable to healthcare accessibility issues for extremely poor OVCs and the resulting inability to get diagnoses; however, this was inconsistent with the Tanzania study that found that OVC in a lower socioeconomic status (34).

The study has some limitations; thus, our findings should be interpreted with those in mind. First, the OVC in the study did not represent all OVC found in the general population in the DRC. Instead, the enrollment with HIV clinics in Haut-Katanga and Kinshasa provinces occurred through multiple entry modes, with 8 out of 10 enrolled because the community-level caseworkers had enrolled an HIV+ adult in the same households. So, the findings may not be generalizable to similar OVC populations. Second, data were not always collected about the OVC from the person most knowledgeable. Third, some variables had a large proportion of cases with missing data, so those variables could not be included as covariates in the statistical model. Fourth, the study utilized a retrospective design, so disaggregation desirable by additional socioeconomic characteristics and type of orphan (single, double, which parent was deceased, or distinction between parent/guardian) was not available. Fifth, there is a potential for a household clustering effect due to some cases of multiple children coming from the same households. However, the secondary data did not include a family-level identifier, which would enable us to make that determination. Finally, many of the OVC and guardian characteristics were unreported, perhaps due to social desirability and social stigma associated with those attributes (e.g., child's schooling status, child's disability status). Regardless of these limitations, the study makes an important contribution in that it is the first of its kind in the DRC, and the findings are important for targeted and efficient interventions for OVC. We have included interpretation of non-significant results for variables that are traditionally seen as very important variables in HIV patient research. When there is no statistically significant difference among one or more of these variables, the authors feel that it is important to report these. It allows other researchers to recognize that a patient's status can be impacted regardless of patient characteristics such as age or sex.

## Conclusions

Given the persisting HIV epidemic in Sub-Saharan countries, targeted, data-driven efforts to inform HIV prevention, care, and treatment programs are imperative, particularly in resource-poor countries such as the DRC. The current study describes the profile of OVC in Haut-Katanga and Kinshasa provinces, showing that OVC is a high-risk subgroup that may merit customized and targeted interventions. A notable proportion of these children were HIV+, many of whom may be orphans, and an abnormally high proportion was not in school. OVC whose school enrollment status was unknown were at a greater risk of being HIV+ than other OVC, implying that their enrollment may be unstable due to HIV. Age and sex of OVC were not significantly associated with elevated odds of being HIV+, suggesting that younger age is not as dominant an aspect of vulnerability to HIV infection as the orphan status (37). We also found significant associations among rurality of health zone, household income, and parents'/guardians' HIV status with OVC HIV+ status. Future program priorities in Sub-Saharan countries must include the development of linkages between OVC care and community support and evidence-based research into the effectiveness of cognitive and educational interventions within the OVC populations. These interventions may include direct and indirect programs that assist with financial support, improvement of neurocognitive development, and in-home activities that revolve around daily living activities and developmentally appropriate play.

## Data availability statement

The data analyzed in this study is subject to the following licenses/restrictions: The program-implementing partners required that data be destroyed after publication. The authors do have data until the publication of the article. The authors can facilitate data access if requested with proper permission from the DRC Ministry of Health. Requests to access these datasets should be directed to Lievain Maluantesa, LMaluantesa@fhi360.org.

## Ethics statement

The studies involving human participants were reviewed and approved by Georgia Southern University's Institutional Review Board approved the study under project protocol number H l9260. Written informed consent from the participants' legal guardian/next of kin was not required to participate in this study in accordance with the national legislation and the institutional requirements.

## Author contributions

Conceptualization: GS, GE, LM, KW, EE, BB, AM, AT, and OI. Methodology: GS, GE, and LM. Formal analysis: GS and KW. Investigation: GS. Writing—original draft preparation: GS, LM, GE, and KW. Writing—review and editing: GS, GE, LM, KW, EE, AM, OI, AT, BB, and AM. Supervision and funding acquisition: GS. Project administration: GS and LM. All authors made substantial contributions to this manuscript, with the following areas of specific contributions and have read and agreed to the published version of this manuscript.

## Funding

This research was supported by the President's Emergency Plan for AIDS Relief (PEPFAR) through the Centers for Disease Control and Prevention (CDC) under the terms of grant number 5 NU2GGH002033-02-00. The findings and conclusions in this journal article are those of the authors and do not necessarily represent the official position of the funding agencies.

## Conflict of interest

Author GE was employed by the organization FHI. Authors LM, EE, and AM were employed by the organization FHI 360. The remaining authors declare that the research was conducted in the absence of any commercial or financial relationships that could be construed as a potential conflict of interest.

## Publisher's note

All claims expressed in this article are solely those of the authors and do not necessarily represent those of their affiliated organizations, or those of the publisher, the editors and the reviewers. Any product that may be evaluated in this article, or claim that may be made by its manufacturer, is not guaranteed or endorsed by the publisher.

## References

[1] Thomas T Tan M Ahmed Y Grigorenko EL A. systematic review and meta-analysis of interventions for orphans and vulnerable children affected by HIV/AIDS worldwide. Ann Behav Med. (2020) 54:853–66. 10.1093/abm/kaaa022 32525205 PMC7646155

[2] Exavery A Charles J Kuhlik E Barankena A Koler A Kikoyo L. Understanding the association between caregiver sex and HIV infection among orphans and vulnerable children in Tanzania: learning from the USAID Kizazi Kipya project. BMC Health Serv Res. (2020) 20: 275. 10.1186/s12913-020-05102-y 32245468 PMC7119283

[3] Pufall EL Eaton JW Robertson L Mushati P Nyamukapa C Gregson S. Education, substance use, and HIV risk among orphaned adolescents in Eastern Zimbabwe. Vulnerable Child Youth Stud. (2017) 12:360–74. 10.1080/17450128.2017.1332398 29170681 PMC5679749

[4] Asanbe C Moleko AG Visser M. Parental HIV/AIDS and psychological health of younger children in South Africa. J Child Adolesc Ment Health. (2016) 28:175–85. 10.2989/17280583.2016.1216853 27562004

[5] PEPFAR. Democratic Republic of the Congo Country Operational Plan (COP) 2019 Strategic Direction Summary. In:Relief PsEPfA, ed. Washington, DC: US Department of State (2019).

[6] Joint United Nations Programme on HIV/AIDS. UNAIDS Terminology Guidelines 2015. Geneva: UNAIDS (2015).

[7] Number of Children Orphaned or Left Unaccompanied by Ebola in the Democratic Republic of the Congo Rising Fast. Geneva: UNICEF (2019).

[8] Federal Ministry of Women Affairs and Social Development (Nigeria). Key Findings Situation Assessment and Analysis on OVC in Nigeria, 2008. In. Nigeria: Federal Ministry of Women Affairs and Social Development (2008).

[9] Bamgboye EA Odusote T Olusanmi I. School absenteeism among orphans and vulnerable children in Lagos State, Nigeria: a situational analysis. Vuln Child Youth Stud. (2017) 12:264–76. 10.1080/17450128.2017.1325545

[10] NIH. The NCI's Dictionary of Cancer Terms. National Cancer Institute (2022). Available online at: https://www.cancer.gov/publications/dictionaries/cancer-terms/def/social-support. (accessed March 23, 2022).

[11] Nichols J Paintsil E Steinmetz A. Impact of HIV-status disclosure on adherence to antiretroviral therapy among HIV-infected children in resource-limited settings: a systematic review. AIDS Behav. (2017) 21:59–69. 10.1007/s10461-016-1481-z 27395433

[12] Sarkar S Corso P Ebrahim-Zadeh S Kim P Charania S Wall K. Cost-effectiveness of HIV prevention interventions in Sub-Saharan Africa: a systematic review. E Clin Med. (2019)10:10–31. 10.1016/j.eclinm.2019.04.006 31193863 PMC6543190

[13] Nsagha DS Bissek AC Nsagha SM. The burden of orphans and vulnerable children due to HIV/AIDS in Cameroon. Open AIDS J. (2012) 6:245–58. 10.2174/1874613601206010245 23248738 PMC3514708

[14] Punpanich W Gorbach PM Detels R. Impact of paediatric human immunodeficiency virus infection on children's and caregivers' daily functioning and well-being: a qualitative study. Child Care Health Develop. (2012) 38:714–22. 10.1111/j.1365-2214.2011.01301.x 21851376 PMC3392445

[15] Sekgoka BM Mothiba TM Malema RN. The socio-economic impact of HIV/AIDS on infected individuals in the Capricorn District of Limpopo Province, South Africa. Af J Phy Health Edu Rec Dance. (2013)19:541–54. 10.10520/EJC142303

[16] Ammon N Mason S Corkery JM. Factors impacting antiretroviral therapy adherence among human immunodeficiency virus-positive adolescents in Sub-Saharan Africa: a systematic review. Public Health. (2018) 157:20–31. 10.1016/j.puhe.2017.12.010 29501984

[17] PNLS. National Guide to Disclosure of Serological Status in Children and Adolescents Infected With HIV in DRC. Democratic Republic of Congo, Ministry of Health, National Program to Fight HIV and STIs (PNLS) (2017).

[18] Haberer JE Cook A Walker AS. Excellent adherence to antiretrovirals in HIV-positive Zambian children is compromised by disrupted routine, HIV nondisclosure, and paradoxical income effects. PLoS ONE. (2011) 6:e18505.. 10.1371/journal.pone.0018505 21533031 PMC3080873

[19] Project SOAR. Meeting the needs of orphans and other vulnerable children: learnings from project SOAR. Project SOAR Summary Brief. Washington, DC: Population Council (2019).

[20] Worldwide SO. Orphan Crisis in DR Congo. Serving Orphans Worldwide. (2019). Available online at: https://soworldwide.org/flames-of-love-dr-congo/ (accessed May 11, 2020).

[21] Bank TW. Democratic Republic of Congo. The World Bank Group. (2020). Available online at: https://www.worldbank.org/en/country/drc/overview (accessed July 15, 2020).

[22] Conserve DF Eustache E Oswald CM. Maternal HIV illness and its impact on children well-being and development in Haiti. J Child Fam Stud. (2015) 24:2779–85. 10.1007/s10826-014-0081-7 28154475 PMC5283390

[23] Doku PN Dotse JE Mensah KA. Perceived social support disparities among children affected by HIV/AIDS in Ghana: a cross-sectional survey. BMC Public Health. (2015) 15:538. 10.1186/s12889-015-1856-5 26048140 PMC4457987

[24] Katana PV Abubakar A Nyongesa MK. Economic burden and mental health of primary caregivers of perinatally HIV infected adolescents from Kilifi, Kenya. BMC Public Health. (2020) 20:504. 10.1186/s12889-020-8435-0 32299411 PMC7161196

[25] Puhr R Heinze G Nold M Lusa L Geroldinger A. Firth's logistic regression with rare events: accurate effect estimates and predictions? Stat Med. (2017) 36:2302–17. 10.1002/sim.7273 28295456

[26] StataCop LLC. Stata Statistics/Data Analysis 15.0, Special Edition. College Station, Texas. Available online at: http://www.stata.com

[27] Ministry of Planning and Monitoring of the Implementation of the Revolution of Modernity (MPSMRM) Ministry of Public Health (MSP) and ICF International. Demographic and Health Survey in the Republic Democratic Republic of Congo 2013–2014. Rockville, Maryland: MPSMRM, MSP and ICF International (2014).

[28] Peer A. Global poverty: Facts, FAQs, and how to help. World Vision. (2021). Available online at: https://www.worldvision.org/sponsorship-news-stories/global-poverty-facts (accessed March 22, 2022).

[29] United Nations Children's Fund Democratic Democratic Republic of Congo. Education - Democratic Republic of Congo. Unicef.org. (2022). Available online at: https://www.unicef.org/drcongo/en/what-we-do/education (accessed March 22, 2022).

[30] The United Nations Educational Scientific and Cultural Organization. Democratic Republic of the Congo - Education and Literacy. Uis.unesco.org. (2022). Available online at: http://uis.unesco.org/en/country/cd. Published 2022 (accessed March 22, 2022).

[31] Li N Tan M Thuma P Grigorenko E. Pediatric symptom checklist-17. Eu J Psychol Assess. (2022) 3:707 10.1027/1015-5759/a000707 PMC10361684 37485035

[32] Ngure K Baeten JM Mugo N. My intention was a child but I was very afraid: fertility intentions and HIV risk perceptions among HIV-serodiscordant couples experiencing pregnancy in Kenya. AIDS Care. (2014) 26:1283–7. 10.1080/09540121.2014.911808 24779445 PMC4087054

[33] Evangeli M Greenhalgh C Frize G Foster C Fidler S. Parenting considerations in young adults with perinatally acquired HIV. AIDS Care. (2014) 26:813–6. 10.1080/09540121.2013.857755 24266514

[34] Bajaria S Exavery A Toroka N Abdul R. Poor linkage to care for HIV-positive OVC with disabled caregivers: a longitudinal study in Tanzania. BMC Public Health. (2021) 21:365. 10.1186/s12889-021-10415-6 33593313 PMC7887816

[35] Exavery A Charles J Barankena A. ART use and associated factors among HIV positive caregivers of orphans and vulnerable children in Tanzania. BMC Public Health. (2020) 20:1251. 10.1186/s12889-020-09361-6 32807138 PMC7433360

[36] Coard E Oliver D Monday F HIV. outcomes within the context of orphans and vulnerable children programing: the 4Children project in South Sudan. BMC Infect Dis. (2022) 22:186. 10.1186/s12879-022-07172-1 35209860 PMC8867614

[37] Belinda P Wanjala G Riechi DA. Influence of Undugu basic education programme on access to basic education for vulnerable out of school children in Nairobi County. Int J Humanit Soc Sci. (2021) 26:26–33. 10.9790/0837-2604082633

